# Big data management skills: accurate measurement

**DOI:** 10.1186/s41039-018-0071-2

**Published:** 2018-05-18

**Authors:** Elspeth McKay, Marlina Binti Mohamad

**Affiliations:** 1 RMIT University, School of Business Information Technology and Logistics, GPO Box 2476, Melbourne, Victoria 3000 Australia; 2 Department of Engineering Education, Faculty of Technical and Vocational Education, Universiti Tun Hussein Onn Malaysia, 86400 Parit Raja, Batu Pahat, Johor Malaysia

**Keywords:** Big data management skills, Digital skill development, Cognitive performance measurement, Instructional design, Human-computer interaction, Item response theory, Rasch model

## Abstract

Some say that big data is transforming business and society. This can mean wide-reaching disruption for commerce, health and world governance. Few authors agree on what constitutes big data, depending on the philosophical stance taken. Our propensity for keeping data archived is posing major issues globally, with retrieval and application of such data crossing ethical boundaries. However, one of the more pressing issues is the growing need for confirming whether those working with such big data have the required digital skills to cope. This paper presents one effective and efficient way to identify such digital skill acquisition. We show the progression from the earlier approach used for measuring proficiency between novice and experienced programmers using traditional statistical measures, to adopting a more comprehensive unidimensional scale that empowers comprehension of human performance and test item performance relative to each other. This methodology offers an effective tool for understanding individual of differences in digital skill development.

## Review

### Introduction

There is no surprise that big data is now receiving considerable attention, especially from the business community (Marr 2016a), where astute entrepreneurs are led to believe they can capitalise on such data to improve their commercial activities. The emerging awareness that is bringing forward a plethora of views from all corners of the digital spectrum has been facilitated by the spread of wireless communication (Alsharif and Nordin 2017). Consequently, there are various opinions of how to define big data. On the one hand, high-end technologies combine information decision-making along with analytic perception gained from examining data through sophisticated technologies that reveal insights from combinations of diverse data sources (Marr 2016b). Others believe the term describes analysis of messy data and use the term to describe the process of finding patterns of data behaviour amongst the conceptual messiness (Melendez 2015; Schöch 2013). While another view identified 10 characteristics of big data that reveal non-linear interrelationships through a three-level framework: fundamental (volume, velocity, variety and veracity), technological (intelligence, analytics and infrastructure) and socio-economic (service, value and market) (Sun 2018). These characteristics require high-level digital skills to manage the information communications technology (ICT) tools involved (Hartley 2003).

Therefore, it does seem natural to link human informational wants and needs to large data sets; to this end, the healthcare industry has acquired a strong presence, where computational analyses can reveal previously unnoticed patterns, trends and associations (Farrington 2016). For example, the recent increase in wearable technologies has enabled collection of health-related real-time data that can be linked to historical records and stored for later retrieval by individuals, healthcare workers and government agencies. Consequently, investment in information technology (IT) to manage and maintain such big data collections is increasing rapidly (Tambe 2014).

The main purpose of this paper however is to show that over time, the methods for measuring people’s digital skills have evolved from taking a classical test theory (CTT) approach to using a more comprehensive Rasch item response theory (IRT) assessment model.

CTT typically uses common statistical analyses to provide information that relates to the reliability of the test score. For instance, according to Adams and Khoo (1996), this means counting the number of people providing a response to a test item, calculating the percentage of total number of valid responses and generating the Pt-biserial that shows the correlation between a response type and the total raw score achieved on the test and the *p* value that represents the significance of that correlation. In contrast, the Rasch IRT enables a finer grained analysis that measures the relative performance of each test item and the relative performance of the individuals taking the test on a unidimensional scale (Bakkar 2016).

As mentioned before, this paper presents the changing ways researchers have determined whether an individual is capable of such problem-based (digital) reasoning (Anonymous 2012a) in a higher education (HE) institutional setting. To this end, there are three separate research studies presented here. Although each study shared a common desire for measurement of a particular digital skill, the objective of each study was to answer a specific research question. In this paper, we are proposing that it is particularly important to differentiate what an individual might know from what they might not (Anonymous 2015). Therefore, criterion-referenced testing is an effective way to measure performance according to the pre-specified criterion (Griffin and Nix 1991). Hence, this paper describes two such experimental studies to reveal that the Rasch IRT model is the best tool to examine such data (Bond and Fox 2015).

Notwithstanding this, the main consideration is whether people in general have the necessary digital skills (Van Laar et al. 2017) to take advantage of the seductive promises that surround the advent of big data. Accordingly, there has been a growing body of literature concentrating on the need for such digital literacy that hitherto encompassed human-computer interaction (HCI) (Anonymous 2008) through the machine dimension (technical requirements) and the human dimension (social interaction). There has been further interest in defining computer literacy (Mat-Jizat 2012) where an emphasis was placed on the researcher to know how to assess an individual’s digital skills. One such contribution to support Mat-Jizat (2012) has been from the Digital Skills Academy in Dublin (‘Digital Skills Academy launches online degree in integrated digital technology, business, design’ 2015), from which graduates were welcomed by top IT organisations in many countries (Hepburn 2013).

And so, we will commence the discussion for this paper on digital skill development that used problem-solving skill development using abstract programming concepts, with a study that took a traditional classroom approach to determine differences between novice and experienced learners who were trying to acquire computer programming skills (Bagley 1990; Tennyson and Bagley 1991). The study was using CTT to measure the effects of paper-based instructional strategies and subsequent digital knowledge acquisition assessment in the form of problem-solving ability an introductory programming course. We then present the Anonymous (2000a) study, which used portions of the Bagley (1990) study as the foundation to investigate the interactive effects of such paper-based strategies (text-plus-textual metaphors/text-plus-graphical metaphors) and cognitive style preferences on the learning outcomes (problem-solving), using the Rasch IRT cognitive measurement model. The Anonymous (2000a, 2000b) work was then taken up by Anonymous (2012a) to investigate the instructional strategies in an online/digital context, also using the Rasch IRT model.

The novelty of this research is the application of the Rasch IRT model to identify a digital skill development framework.

### Study 1: problem-solving abstract concepts

In keeping with the Sun (2018) stance on big data characteristics and the non-linear interrelationships through the ‘technological framework’ (intelligence, analytics and infrastructure), the Bagley (1990) study represents the initial instructional design (ID) pedagogy that was used to underpin other IS-doctoral theses (Anonymous 2000a, 2012a, 2012b, 2016a, 2016b). Digital skill development was a common pedagogy focus for each of these theses. This paper proposes that such digital skill development is a key attribute for dealing with big data management, earlier described by Sun (2018) as the 10 big characteristics. The Bagley thesis was entitled ‘Structured Versus Discovery Instructional Formats for Improving Concept Acquisition by Domain-Experienced and Domain-Novice Adult Learners’. The purpose of this research was to investigate the interaction of instructional strategies with students that had no prior domain knowledge of computer programming, on the assumption that learners with no such knowledge would learn programming concepts better with a structured strategy versus those who had to construct the necessary conceptual knowledge (Tennyson and Bagley 1991, p. 2) The Bagley research involved paper-based self-paced instructional booklets (structured format/discovery format) that commenced with a brief review of the introductory material, which then led the students through the instructional strategy.

There was expository content for each learning goal, such as definitions, best example and a range of other examples, non-examples and worked examples of problems, according to the ID principles for learning abstract concepts (Merrill et al. 1992).

The structured instructional booklet involved three lessons: data types, programme structure and three PASCAL statements (Readln, Writeln and assignment), and writing new PASCAL programmes. The discovery booklet involved four parts: basic PASCAL programmes (with sample data), three problem statements (with input, output and the algorithmic process), pseudocode example and three problem statements.

#### Self-paced instructional booklets for the exploratory study

In the structured instructional booklet, the initial directions were to proceed through the three lessons to learn about basic PASCAL programming; in the discovery instructional booklet, learners were encouraged to discover the solutions to the problems using their past knowledge or relevant content provided in the structured booklet.

The learning content reflected the problem-solving programming conventions of the time for developing algorithms and pseudocode (or structured English statements). This instructional material involved sequence, selection and repetition logic patterns (or programming control structures). The generic textual instruction was designed to guide learners through progressively more complex content (Bagley 1990). Following the expository learning content, there was a four-page interrogatory section that led the learner through the instructional material and to complete the programming problems. Intentional instructional gaps were left in the practice examples to encourage the learner to write parts of the algorithms on their own. The intention of the strategy was for the learner to gradually create more of the algorithm, with less information given as the task progressed. This was known as a fading technique and according to Bagley (1990) was shown to be more successful for novice programmers than for experienced programmers.

Instruction commenced with a description of an algorithm. Then, the learners were guided by an epitome statement that gave the instructional goals and objectives for each sub-lesson, relating to four sub-lessons (Anonymous 2000a):

- Logic patterns: (sequential, repetition and conditional control structures)
- Repetition logic: (using the DOWHILE and the REPEAT .. UNTIL structures)
- Comparison of the two logic patterns
- Example problems

Following the last example problem, a short review or summariser of the four sub-lessons was given, followed by an expanded epitome statement, devised to lead the learner to the next part of the lesson. Then, there were two example problems that were fully explained in the instruction, including the following:

- An objective (setting out the expected instructional outcome)
- The situation description (setting the scenario context)
- The task description that involved a six-point suggested solution.

#### Procedural knowledge development strategy

Procedural knowledge provides the pathway to the instructional outcome, which in this case is to write an algorithm that represents the digital solution for solving a particular problem. For example:

- Redefine the problem (in terms of input/processing/output)
- Logic patterns (control structures required to support the solution)
- The repetition question (most novice programmers were unaware of the need for conceptualising this component)
- Repetition starting point (once again novice programmers found this difficult)
- Repetition conditional statement
- Alternate repetition condition
- A suggested solution algorithm

To reinforce the declarative, procedural and conceptual knowledge (Bagley 1990), example problems were followed by two more practice problems. Declarative knowledge relates to the description of things, events or processes and their associated relationships; procedural knowledge relates to knowing how to perform the necessary tasks; conceptual knowledge requires an understanding of the explicit/implicit principles involved. The learning task section was presented as an interrogatory learning format (Anonymous 2000a). Suggested solutions were given for the first practice problem (Anonymous 2000a). However, in the second practice problem, the final task of writing a complete solution algorithm was left blank for the participant to finish; hence, ‘leaving gaps in this manner provides learners with an opportunity to practise their declarative knowledge’ (Anonymous 2000a). By working through the problem examples, participants could check their procedural and conceptual knowledge against the suggested solutions. This technique was known as a coaching technique, which pointed out key issues relating the problem to the definition, best examples and non-examples, according to Bagley (1990).

#### Research design

This study involved a 2 × 2 factorial design to test the independent variables, prior knowledge and instructional format (structured/discovery), and to test novice and experienced adult learners’ computer programming knowledge acquisition. A Wechsler Adult Intelligence Scale (WAIS) arithmetic reasoning test was given to all learners prior to the start of the experiment; the results were used as a covariate to determine whether arithmetic reasoning might affect the pre-test (a screening test for prior computer programming knowledge) and the post-test (to determine level of change in knowledge acquisition).

#### Methodology

There were 120 students who were randomly assigned to four treatment groups (novice or experienced programmers: structured instructional format; novice or experienced: discovery instructional format). Novice programmers were deemed as having no prior knowledge of any computer programming language, while the experienced programmers had a minimum of one computer programming course in BASIC. The dependent variables were the correct scores achieved on the post-test. Analysis of variance (ANOVA) was used to analyse the results.

#### Results

Overall, the Bagley (1990) results confirmed two hypotheses: (1) that given a structured instructional format, students with no prior knowledge of computer programming improved their learning of computer programming and (2) that giving a discovery instructional format to students who did have prior computer programming knowledge did improve their learning of a new computer programming language. Yet, the differences in the results when considering the interactive effect of instructional strategy and prior domain knowledge (experienced/novice) on performance outcomes revealed that given the structured instructional format, the experienced learners did not perform as well. And when given the structured instructional format, the novice programming learners who would not be using any relevant prior knowledge outperformed the experienced learners given the same treatment.

#### Discussion

These instructional strategies provided a general insight into how researchers investigated the acquisition of problem-solving skills to solve abstract problems (and more specifically for writing computer programs). At the time of this study and long before the concept of big data was conceived, researchers were using the term ‘computer literacy’ to describe the HCI. Then, the research focus was mainly on the cognitive processing styles (structured/discovery) and peoples’ capabilities expressed as arithmetic reasoning (problem-solving) abilities. We maintain that it was the advent of Web 2.0 and the introduction of more powerful computers that has advanced the installation of our big data platforms and has refocused educational researchers’ view of how to prepare effective instructional strategies to enhance the digital skill development that is necessary to deal with such big data.

### Study 2: paper-based instructional metaphor format and cognitive style

Through the 1990s, researchers were looking at ways to individualise instructional materials; while at the same time, computer systems were affording easier adoption of graphical content to provide people’s informational needs. In support of this assertion, the Anonymous (2000a) study drew on the Bagley (1990) work to include an investigation of the interactive effects of instructional format involving textual/graphical metaphors and cognitive style (Anonymous 2000a) for the acquisition of introductory programming/problem-solving techniques.

Again, and in keeping with the Sun (2018) stance on big data characteristics and the non-linear interrelationships through the ‘technological framework’, described as intelligence (often referred to as artificial intelligence) and data-driven analytics involving collection, organising to discover visual patterns (to support decision making) (Sun 2018, p. 5); this research presented a novel approach for dealing with the cognitive assessment. To this end, the researcher investigated the interactive effects of instructional mode (text/graphical metaphors) with a student’s individual cognitive instructional preference (verbal/imager). It is further proposed here that these personal instructional characteristics may have some bearing on an individual’s ability to deal with the high-level nature of the digital skills that are necessary to manage big data. The research involved pursuing the following question in a paper-based experimental context: *does the interaction of instructional format and cognitive style construct affect the acquisition of abstract programming concepts?*

**Fig. 1:**
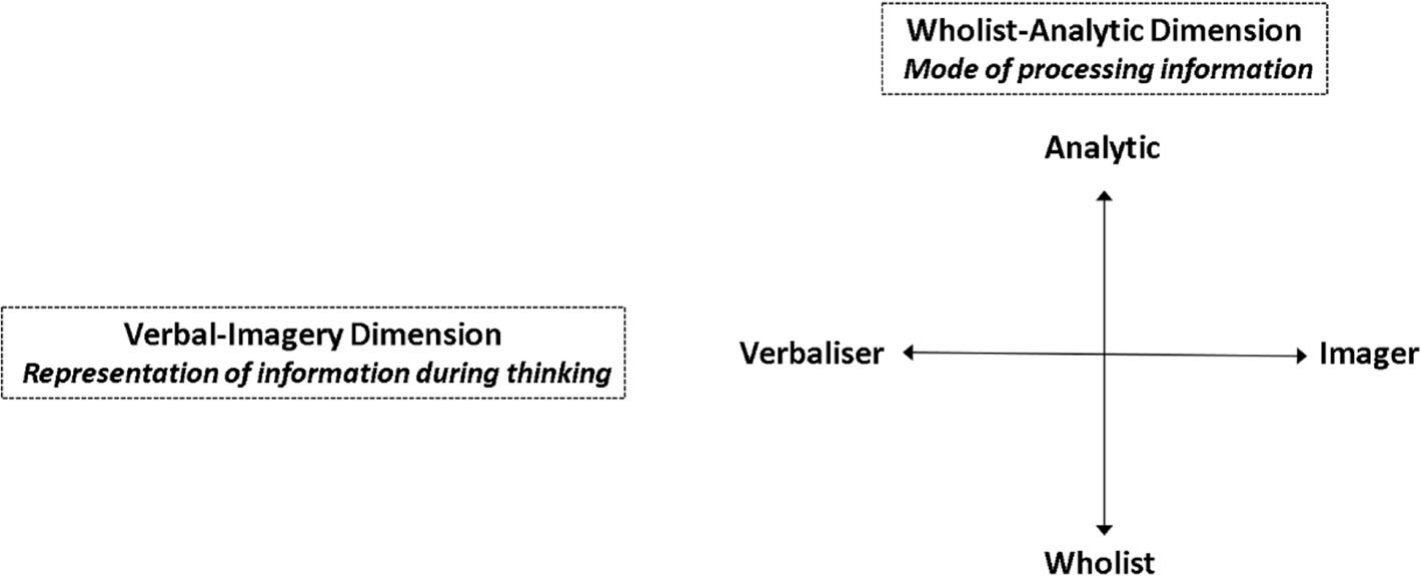
Riding’s cognitive style continua (Anonymous 2000a). This figure represents the cognitive style model described by Richard Riding and Stephen Rayner in 1998 identifying an individual’s position on both orthogonal continua. An individual’s position on one dimension is independent of their position on the other. The wholist-analytic dimension describes whether an individual tends to process information in wholes or parts, while the verbal-imager dimension describes whether an individual is inclined to represent information verbally or visually during thinking (Riding and Rayner 1998)

As described in more detail in Anonymous (2000b), cognitive style has been categorised as wholist-analytic and verbal-imager where the cognitive continua form an integrative model of cognitive style (Riding and Rayner 1998). Figure 1 shows this cognitive style model, which can identify an individual’s position on both orthogonal continua, according to Riding (1993). And so, an individual’s position on one dimension is independent of their position on the other (Riding 1993). Accordingly, the wholist-analytic dimension describes whether an individual tends to process information in wholes or parts, while the verbal-imager dimension describes whether an individual is inclined to represent information verbally or visually during thinking.

**Fig. 2:**
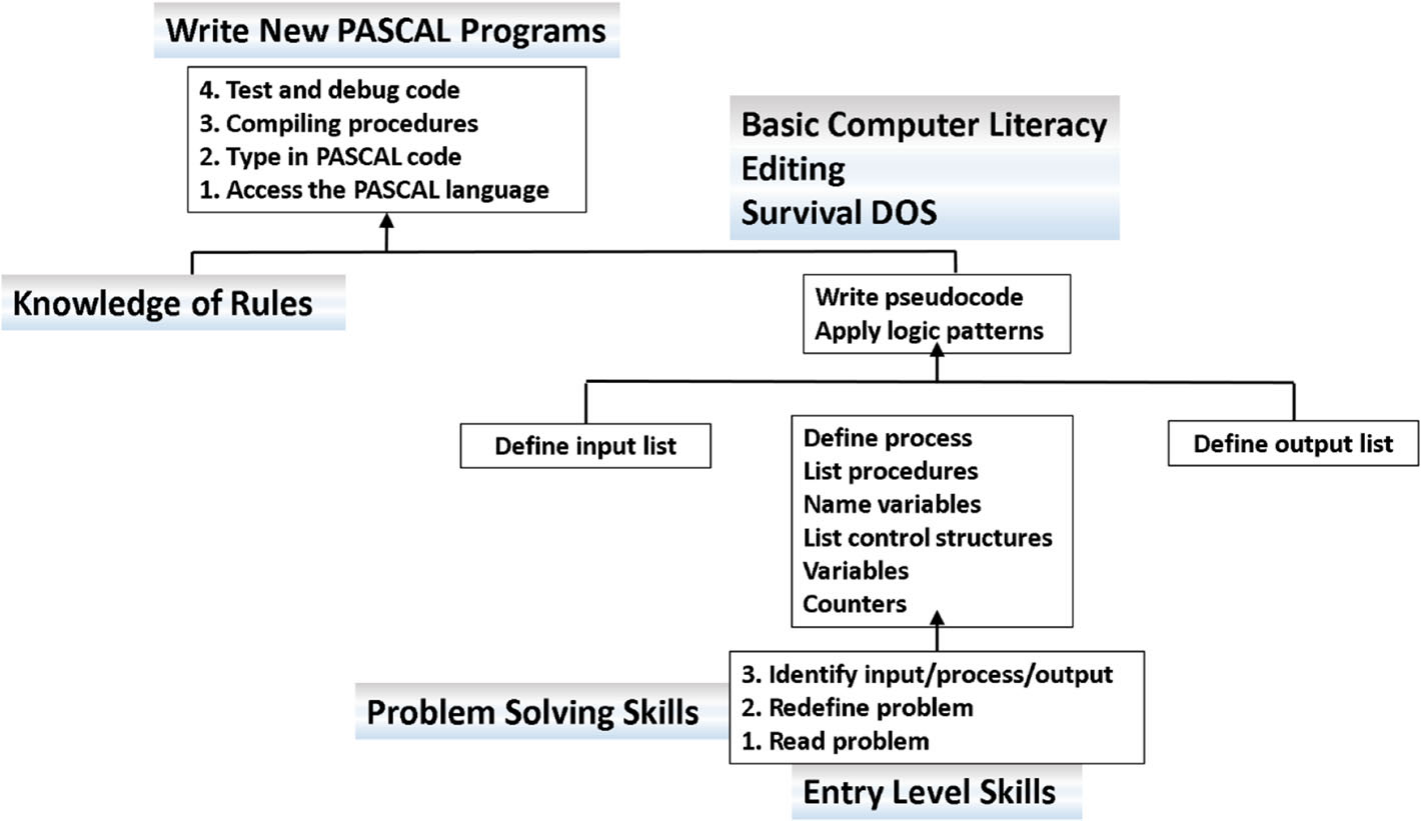
Problem-solving hierarchy (Anonymous 2000a). This figure represents the strategic programming knowledge required for writing a new PASCAL programme. Instructional modules provide the anticipated instructional outcomes required to demonstrate strategic programming knowledge. Commencing from the bottom of the figure they include the entry-level skills, basic computer literacy; problem-solving, redefining the problem to prepare an algorithm; and knowledge of the programming language rules. Each module identifies a sequence of tasks necessary to achieve the instructional outcome. The learning hierarchy is described in full in the Anonymous 2000 thesis—see from page 158

**Fig. 3:**
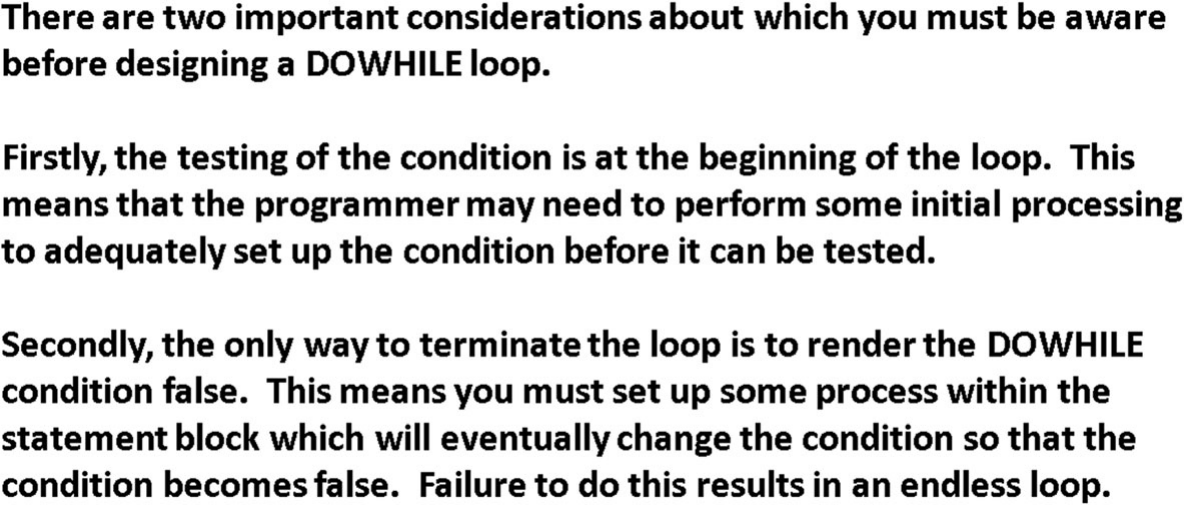
Text-plus-textual metaphor (T1)—repetition programming logic (Anonymous 2000a). This figure represents a textual metaphor for a ‘do while loop’

**Fig. 4:**
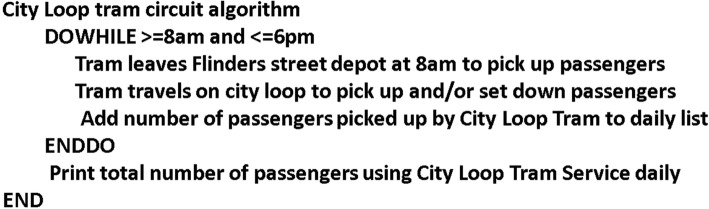
Text-plus-graphical metaphor (T2)—repetition programming logic (Anonymous 2000a). This figure represents an example of a text-plus-graphical metaphor for a ‘do while loop’

**Fig. 5:**
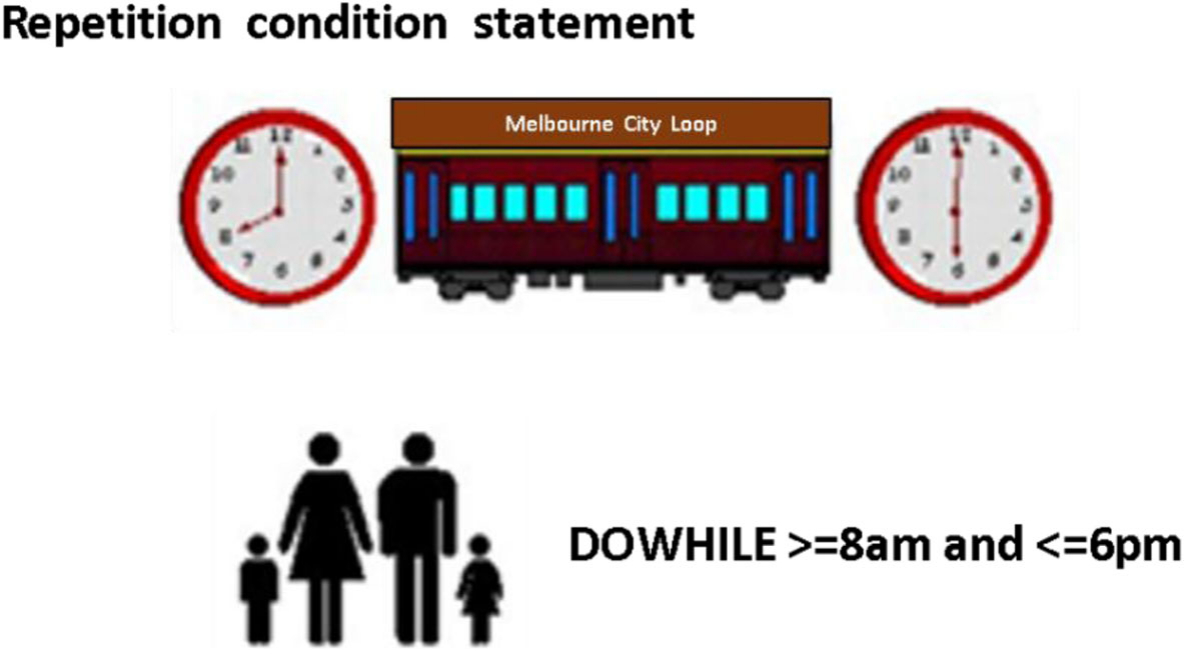
QUEST variable map (Anonymous 2000a). This figure shows how the QUEST estimate develops a uni-dimensional (logit) scale (− 3.0 to 1.0) with equal intervals along each axis as it measures participants’ performances and test items together. The *x*’s on the left hand side of the figure represent an individual participant’s performance with the total number of participants being 195. On the right hand side of the figure is the difficulty rating of each test item’s performance (partial credit scored test items have multiple entries: 8.1, 8.2 and 9.3, 20.2)

The Anonymous (2000a) study reduced the Bagley (1990) content to concentrate solely on the programming logic patterns that would be teachable in a one-hour practical tutorial session. There were three paper-based instructional booklets used by groups of participants. As pre-instructional material, the first self-paced booklet was used by all the participants and contained the preliminary expository instructional content as an introduction to computer programming (Fig. 2). The second and third booklets were designed in conjunction with the experiment to examine the interactive effect of instructional format: text-plus-textual metaphor (T1 Fig. 3) or text-plus-graphical metaphor (T2 Fig. 4) programming solution metaphors in terms of declarative, procedural and conceptual knowledge (Fig. 5).

In order to write a computer programme to solve (in a digital sense) a particular problem, Fig. 2 shows the complete task hierarchy as entry level skills, problem-solving skills, knowledge of (digital) rules and basic computer literacy. The entry-level skills defined here as problem-solving skills, include such tasks as being able to read and redefine the problem, then identifying the data-input and the (digital) processing necessary to achieve the data output. The Anonymous (2000a) research concentrated on the problem-solving skills component of Fig. 2 to situate the instructional strategies for writing pseudocode for the algorithm.

**Fig. 6:**
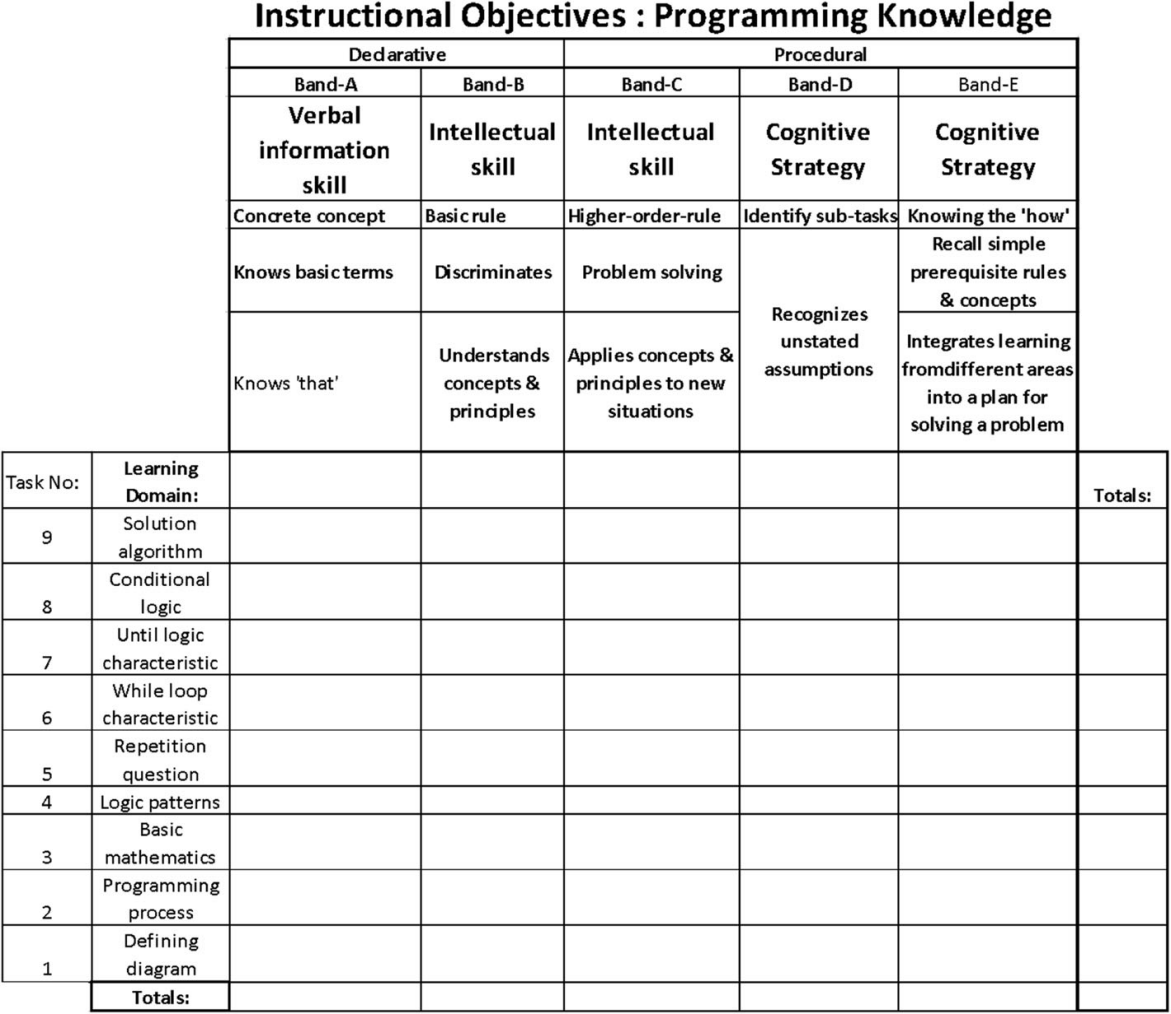
QUEST fit map (Anonymous 2000a). This figure shows the fit statistics (listed horizontally .56 to 1.40 is the infit mean square); the asterisks represent the magnitude of the fit statistic for the test item on the same line. The test items that fall between the two vertical dotted lines (thresholds .77 to 1.30) are considered acceptable; test items to the left overfit (see test item 34), indicating duplication or having limited contribution. Underfit test items to the right of the threshold lines, measure something else and need rewording

Figure 6 provides an example of how the instructional strategy conveyed the expository information for expressing the control flow statement known as a ‘do while loop’.

The textual metaphors (Fig. 3) were used to expand Bagley’s expository instructional strategy. In so doing, they articulated the critical attributes of the concept-to-be-learned according to the first principles of instruction (Merrill 2009). In most cases, the textual metaphor was used after the generic textual description.

With the text-plus-graphical metaphor instructional treatment, the instructional format involved the same lesson on programming logic patterns as the text-plus-textual metaphor format booklet. However, the textual metaphor was replaced with a graphical representation (Fig. 4). The graphics were selected to represent the textual metaphors and chosen by their commonly recognisable and distinguishing (or salient) features.

A ‘test instrument specification matrix’ was devised to design the test items that would be used to determine the expected introductory programming knowledge acquisition. The horizontal axis was used to depict the instructional objectives, with the vertical axis used for the programming content (or learning domain). As shown in more detail in the (Anonymous 2000a) thesis, the programming of such logic patterns was defined as a significant subdivision of programming skill. The learning domain is shown here (Fig. 5) as a continuum, beginning with simple programming concepts at one end, developing into more complex conceptual programming tasks at the other. There were nine categories of digital skill identified along this continuum. The instructional objectives consisted of two categories of specific programming knowledge.

Declarative knowledge was divided into two levels of skill (Anonymous 2000a):

1. Verbal information (knowing isolated rules)
2. Intellectual (knowing how to discriminate between concepts and principles)

The procedural knowledge was divided into three levels:

1. Intellectual skill (higher-order-rules for problem-solving)
2. Cognitive strategy (recognising sub-tasks)
3. Ability to integrate learning across learning domains (for implementing a comprehensive plan of action)

#### Validating the learning content

Learning content validity testing was performed on the pre-test instrument and confirmed by independent content specialists. To determine scoring range for levels of prior domain knowledge, variance on the pre-test was established in a pilot study: a pre-test score of < 28% for novice-programmers, while the experienced-programmer group’s score was recorded as ≥ 28% from the data obtained. The lower reliability recorded by the experienced programming group was related to the higher pre-test scores, reflecting less change between the pre- and post-test scores. These pre-test results supported the Bagley (1990) thesis. Accordingly, the pre-test met the original objective of dividing the learners who passed the pre-test after taking a programming course and were, therefore, considered experienced programmers (Anonymous 2000a). At the core of this methodology was the initial instrument validation process which must be followed to ensure that there was internal consistency and that a reliable measurement scale had been established. This means that the researcher must ascertain that test items were a fit of the Rasch IRT model, which is discussed in more detail further on in this paper.

#### Research design

The experiment employed a 2 × 2 factorial design. As the extent of a design effect was unknown, simple random sample statistics were not used. Since the research questions were interested in the magnitude of effect, ANOVA was considered inappropriate (Izard 1999), and the Cohen (1977) approach was used instead. The Riding and Cheema Cognitive Styles Analysis (CSA) test was conducted before the experiment, so the researcher used another statistical measurement tool that was better suited to analyse the test data for the digital skills’ performance (Anonymous 2000a). This tool was the QUEST interactive test analysis system (Wu and Adams 2007), which uses both classical test theory (CTT) and the Rasch model known as the item response theory (IRT) (Griffin and Nix 1991), to evaluate the digital skill development and the test item behaviour on a unidimensional probability scale (Bond and Fox 2015).

#### Methodology

There were 195 participants enrolled in a HE Business IS Computing course who completed the instructional treatments and cognitive assessment. The research schedule involved the participants in a five-stage process, including (1) registration and consent to participate, (2) undertaking the CSA (the resulting CSA-ratio was used to allocate instructional treatment), a pre-test (to determine their prior domain knowledge), the instructional treatment (T1 or T2) and a post-test that was used to determine the level of change in knowledge (digital skill acquisition).

#### Data analysis

**Fig. 7:**
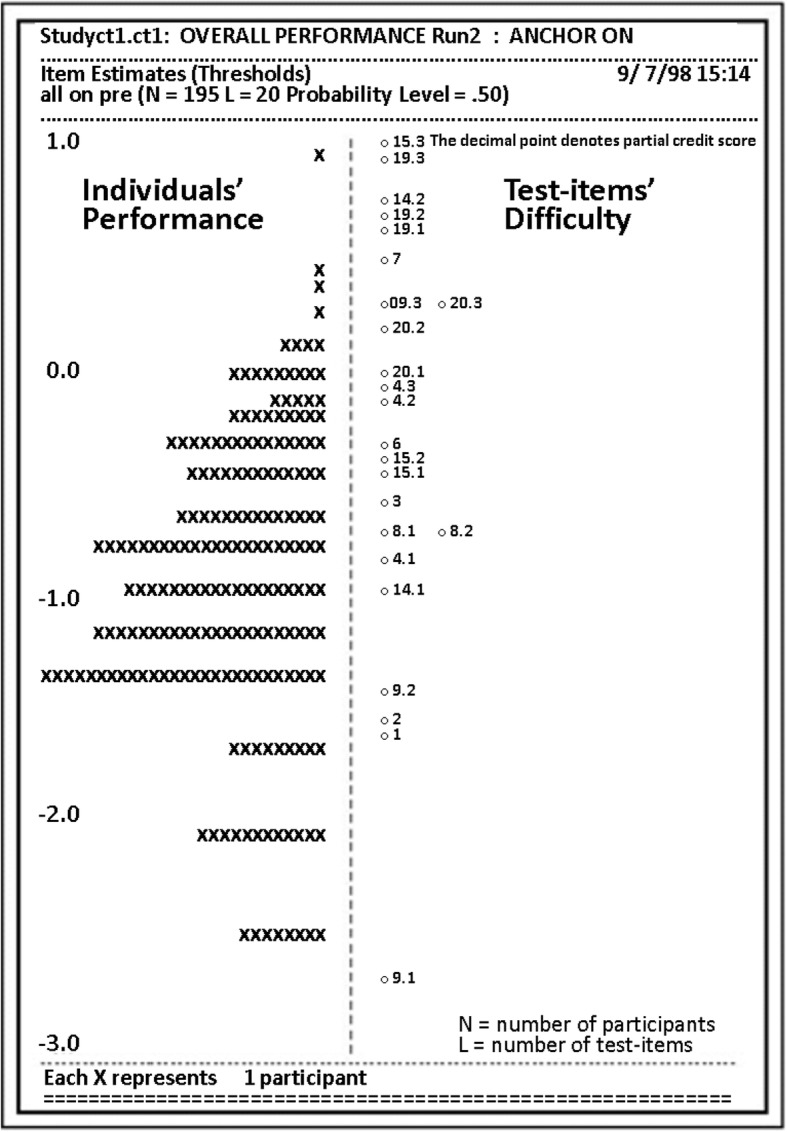
Relative distribution—four groups (Anonymous 2000a). This figure shows the relative distribution of the four-instructional treatment/gender groups (treatment 1—textual metaphor and treatment 2—graphical metaphors). Females given the graphical metaphors achieved the highest post-test distribution. Females with the textual metaphor format had the lowest distribution. The two male groupings had similar distributions, resting between the two female distributions

The data analysis was conducted using the QUEST measurement tool (Adams and Khoo 1996), while the digital skill acquisition was evaluated in terms of the magnitude of change in participant proficiency (magnitude of effect size as defined by Cohen’s statistical power analysis (Cohen 1977)). QUEST allows for improved analyses of an individual’s performance relative to other participants (Anonymous 2000b) and relative to the test item difficulty. On the left side of Fig. 7, each participant or ‘case’ is depicted by an ‘*x*’, and the test items are shown on the right side of the map. The Rasch IRT estimates the probability of an individual making a certain response to a test item (Anonymous 2016).

**Fig. 8:**
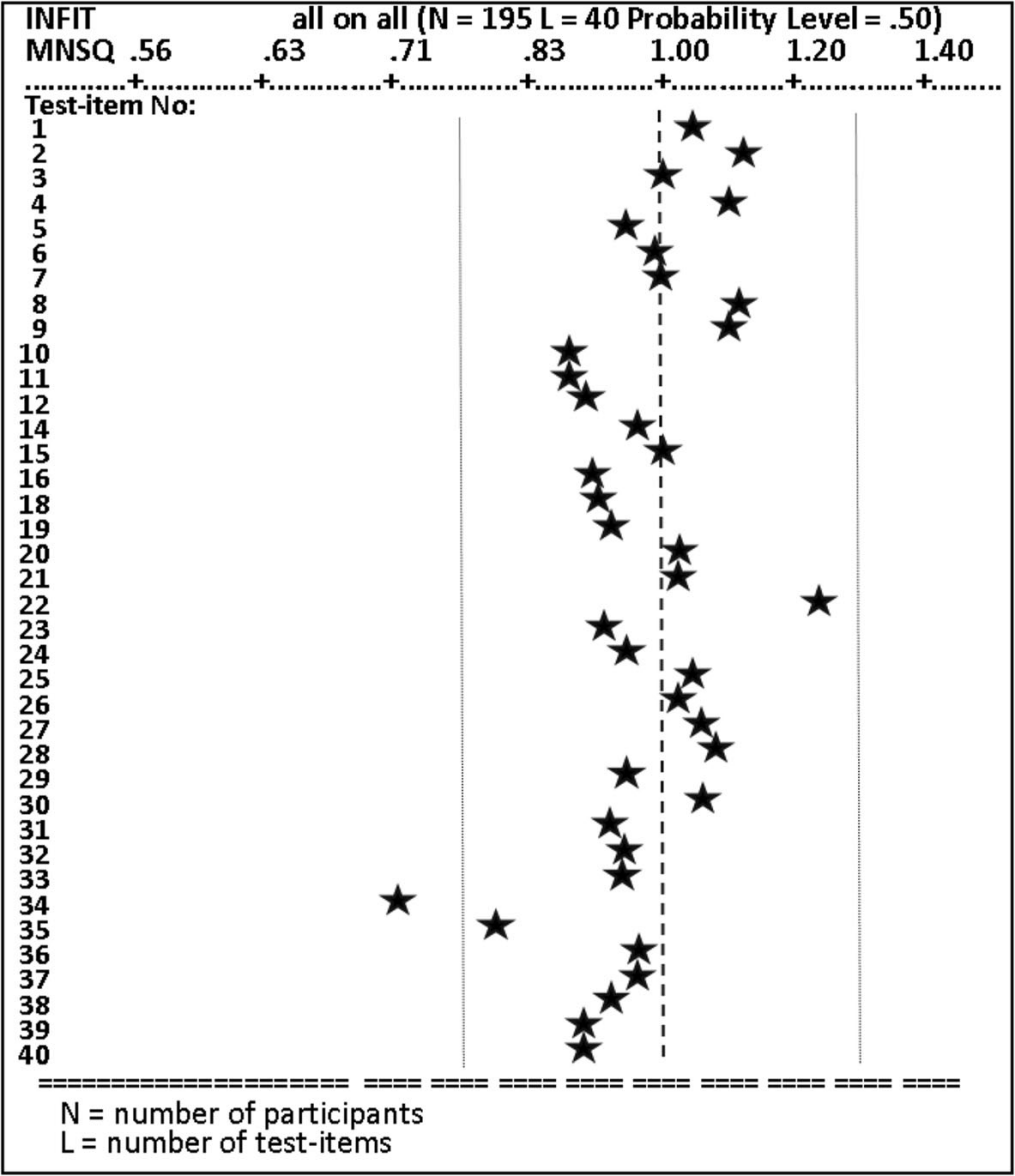
First screen of web-mediated instructional module (Anonymous 2012a). This figure shows the opening web-mediated instructional system’s screen display including how to navigate the instructional content and guidance on how to work through the instructional modules, (knowledge) navigation buttons or hyperlinks, and menu positioning relating to the current topic and learning content

The QUEST Item Fit Map (Fig. 8) provides a visual check of magnitude of the fit statistic for the test items. Overall, these test items fit the Rasch model (Bond and Fox 2015), with the exception of test item 34, which ‘overfitted’ the Rasch model because it fell outside of the left-hand vertical dotted line, indicating a mean square that was 30% below its expected value. Each test item had been designed to test for specific types of digital programming skill/knowledge. In general, the higher the programming knowledge required to correctly answer a question, the higher on the logit scale the test item will appear.

Looking at Fig. 7, it is apparent that this pre-test was relatively easy for 85% of the participants and more difficult for the remaining 15%, who were able to answer the first part of test item 9 but would struggle to achieve test items 1 and 2 and the rest of the instrument.

#### Results

The final results revealed that the verbalisers (measured using the Riding and Cheema (1991) CSA tool) performed best when given the instructional format enhanced with graphical metaphors and that some imagers may respond better with text-only material. Furthermore, when describing the interactive effects of the inherent mode of processing information (wholist/analytic), and instructional format, the ‘wholists’ performed better when given graphical metaphors than when receiving the text-only material. Moreover, when considering this CSA-dimension, the analytics also performed better with the graphical metaphors, although not as well as the wholists did. However, when considering the more detailed analysis of results relating to the combined CSA-dimensions, there was a more complex explanation. It was evident that when given a graphics-enhanced instructional strategy, particular combinations of the cognitive style construct had the potential to perform much better than others. In particular, the largest effect size for the integrated cognitive style groupings was accomplished by the wholist-imagers and analytic-verbalisers, while analytic-imagers perform poorly with the graphics format. This result was as expected from the exploratory study results (Anonymous 2000a) and confirmed in the final experiment.

**Fig. 9:**
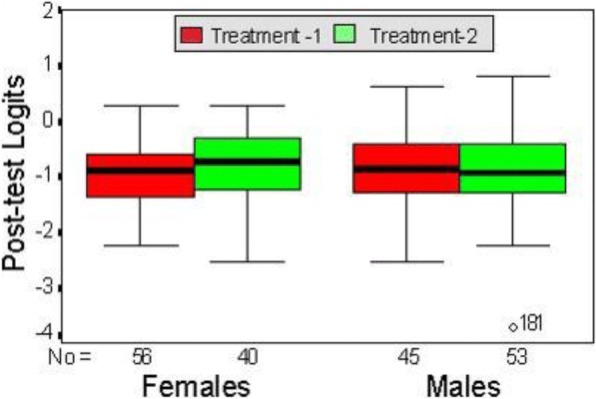
Research schedule (Anonymous 2012a). This figure shows the research schedule comprising the four research study stages: stage 1, day 1—involving the CSA screening test to allocate participants to their instructional treatment; stage 2, day 2—involving the pre-test for prior domain knowledge; stage 3, day 2—involving the experiment; and stage 4, day 2—the post-test

Insofar as the consequence of gender in this study, there was a total of 98 males and 96 females in the final experiment. Of the males, 45 participants were given the textual metaphors and 53-participants the graphical metaphors; whereas 56 females were given the former instructional treatment and the remainder (40) graphical metaphors (Anonymous 2000a). Figure 9 shows the relative distribution of these four groups, with females given the graphical metaphors achieving the highest post-test distribution and females with textual format having the lowest distribution. The two male groupings have similar distributions, resting between the two female distributions. It was therefore determined that the null hypothesis that was being tested indicated that gender would have no impact on the cognitive performance of one treatment group (instructional format/cognitive style compared to another), although females presented with graphical treatment performed better than females with the textual treatment.

The final conclusions drawn from this research study indicated that, when describing the interactive effect of an instructional strategy on the means which individuals think of what they see (the ‘verbaliser-imager’ continuum):

1. Verbalisers perform best when given an instructional format enhanced with graphical metaphors
2. Some imagers may respond better with text-only material

Furthermore, when describing the interactive effects of the inherent mode of processing information and instructional format (the wholist-analytic continuum):

1. Wholists perform better when given graphical metaphors than when receiving the text- only variety
2. Analytics also performed better with the graphical metaphors, although not as well as the wholists

However, a more detailed analysis of results revealed a more complex explanation: it was evident that when given a graphics enhanced instructional strategy, particular combinations of cognitive style construct have the potential to perform much better than others.

In particular, the largest effect size for the integrated cognitive style groupings was accomplished by the wholist-imagers and analytic-verbalisers. And as expected from the exploratory study results and equally confirmed in the final experiment, the analytic-imagers performed poorly with the graphics format (Anonymous 2000a).

#### Summary

This study provided an insight into how researchers can investigate the interactive effect of instructional strategies and cognitive style preferences on learning how to solve (in a digital sense) a particular business-related problem, and more specifically how to develop an algorithm that can be used to write a computer programme. This study was also before the notions of big data implementations, yet researchers were commencing to identify the machine-dimensions of HCI (Anonymous 2008). One limitation of translating the Anonymous (2000a) work to the digital environment was the paper-based pedagogy/environment, whereas Anonymous (2012a) moved the impact of this research into the online (or web-mediated) technologies.

### Study 3: online instruction metaphors and cognitive style

#### Introduction

The Anonymous (2012a) study extended the Anonymous (2000a) research to examine the interactive effects of web-mediated instructional strategies and cognitive preferences in the acquisition of introductory programming concepts in Malaysian universities.

Once again, and in keeping with the Sun (2018) stance on big data characteristics and the non-linear interrelationships through the ‘technological framework’, this thesis extended the novel approach set by Anonymous (2000a) to deal with the cognitive assessment in a digital environment. Whereas the instructional strategies investigated by Anonymous (2000a) were paper-based, and involved graphical metaphors (Anonymous 1999a, 1999b), this study instead interrogated whether ICT elements such as: signals (or cues); interactive animation; navigational tools; words and graphics, influenced students’ cognitive performance, and whether there were interactive effects of their cognitive preferences that may have contributed to the results. Like the paper-based Bagley (1990) and Anonymous (2000a) research, this empirical study examined the performance of novice-learners (or programmers) with different cognitive preferences, only this time the study used two web-mediated instructional strategies: (1) text-plus-textual format and (2) text-plus-graphical format. The research question under examination was *does the interactive effect of web-mediated instructional strategies and a learner’s cognitive style preference affect the acquisition of introductory computer programming concepts?*

The participants were second-year undergraduate students, in Malaysia, who were required to enrol in an Introductory Programming course as part of their educational programme commitment. The results suggested there is no clear evidence that cognitive preference played an important role in cognitive performance when learning from web-mediated instructional modules. However, it was observed that the analytic-verbalisers performed better when the instructional format they received matched their cognitive preferences. Because the participants were novice learners, the influence of prior domain knowledge could not be ignored. The researcher knew the use of high-quality measurement tools to assess the effectiveness of web-mediated instructional strategies was important. The findings suggested guidelines on how to design web-mediated instruction for high-element interactivity knowledge domains such as the acquisition of computer programming concepts.

#### Modified context for adoption online

In selecting appropriate instructional strategies to be implemented in designing instructional systems for introductory programming concepts (and with appropriate permission), the treatment drew on the instructional programme employed by the Anonymous (2000a) work. The Anonymous (2012a) study adapted the web-mediated learning content to focus on C++ instead of the original Bagley (1990) paper-based introductory Pascal programming language manuals that were redesigned by Anonymous (2000a). Consideration was given to several factors such as the learner cognitive preferences, choice of delivery system, task complexity and technology (Merrill, 2009). In considering the learner, the constructivist model assumes that learning occurs at the individual’s level. Thus, Anonymous (2012a) proposed that instruction should be designed to cater for individuals and not for groups of learners. Delivering instruction through web-mediated technology offers individuals the flexibility to access the instruction at their own pace and provides more time for reflection (Fetaji and Fetaji 2007). And so, in terms of learning-task complexity, it has been shown that an introductory programming concept is already complex enough for experienced learners (as it involves high-element interactivity information) let alone for novice learners (Garner et al. 2005); (Anonymous 2012a). Given this level of complexity, the ID must take into consideration that the learners will need to have adequate prerequisite knowledge or skills to acquire more advanced skills or knowledge. Therefore, developing a unit of web-mediated instruction for the Anonymous (2012a) study was based on the Gagné nine events of instruction (Gagné 1985). At that time, researchers were becoming interested in the effectiveness of the internet as an instructional device, for instance, the seven dimensions that contributed towards student success in web-mediated learning environments (Schrum and Hong 2002). These dimensions included access to tools, experience of technology, learning preferences, study habits and skills, goals and purposes, lifestyle factors and personal traits, and learner characteristics. In terms of a learner with different cognitive styles, they had similar but non-linear approaches towards learning in web-mediated environments (Chen 2010). Yet another study revealed that university students preferred structured and concept-based web-mediated instruction to a more traditional classroom approach (Yang and Tsai 2008). Moreover, the Anonymous (2012a) study found that individual preference in web-based learning was influenced by an individual’s ICT experiences. To further examine the effect of web-mediated instruction in terms of ‘instructional strategies’, the learning materials concentrated on both textual and multimedia formats.

The instructional topics within the web-mediated instructional system (WMIS) were developed to establish the possible advantages of integrating web-mediated interactivity with the textual and graphical instructional strategies. These modules were developed in two versions in the web-mediated environment according to the two Anonymous (2000a) paper-based treatment booklets that comprised textual and graphical metaphors. The Anonymous (2012a) WMIS topics were represented as text-plus-textual metaphor (T1) and the text-plus-graphical metaphor (T2) instructional format. The learning content adapted the Pascal content of the Anonymous (2000a) instructional materials to the C++ content in a web-mediated instructional environment. The instruction was also developed according to the regular lesson plan implemented at the Malaysian university. Moreover, the newly developed Anonymous (2012a) instructional strategies also included programming concepts and examples of C++ programming (Deitel and Deitel 2008; Malik 2008; Zak 2008).

Although at the time the user-interface design was known to be one of the critical factors in successful web-mediated instruction, typically the instructional designers or educational software developers often neglected usability issues. Anonymous felt that if they did pay attention to usability issues, students relied on intuition and experience rather than theory-based models, so the Anonymous (2012a) user-interface design took this issue into account. As such, it facilitated the learners to focus on the instructional strategies, thereby reducing the mental effort required for navigating their way through the modules (Lohr 2000).

**Fig. 10:**
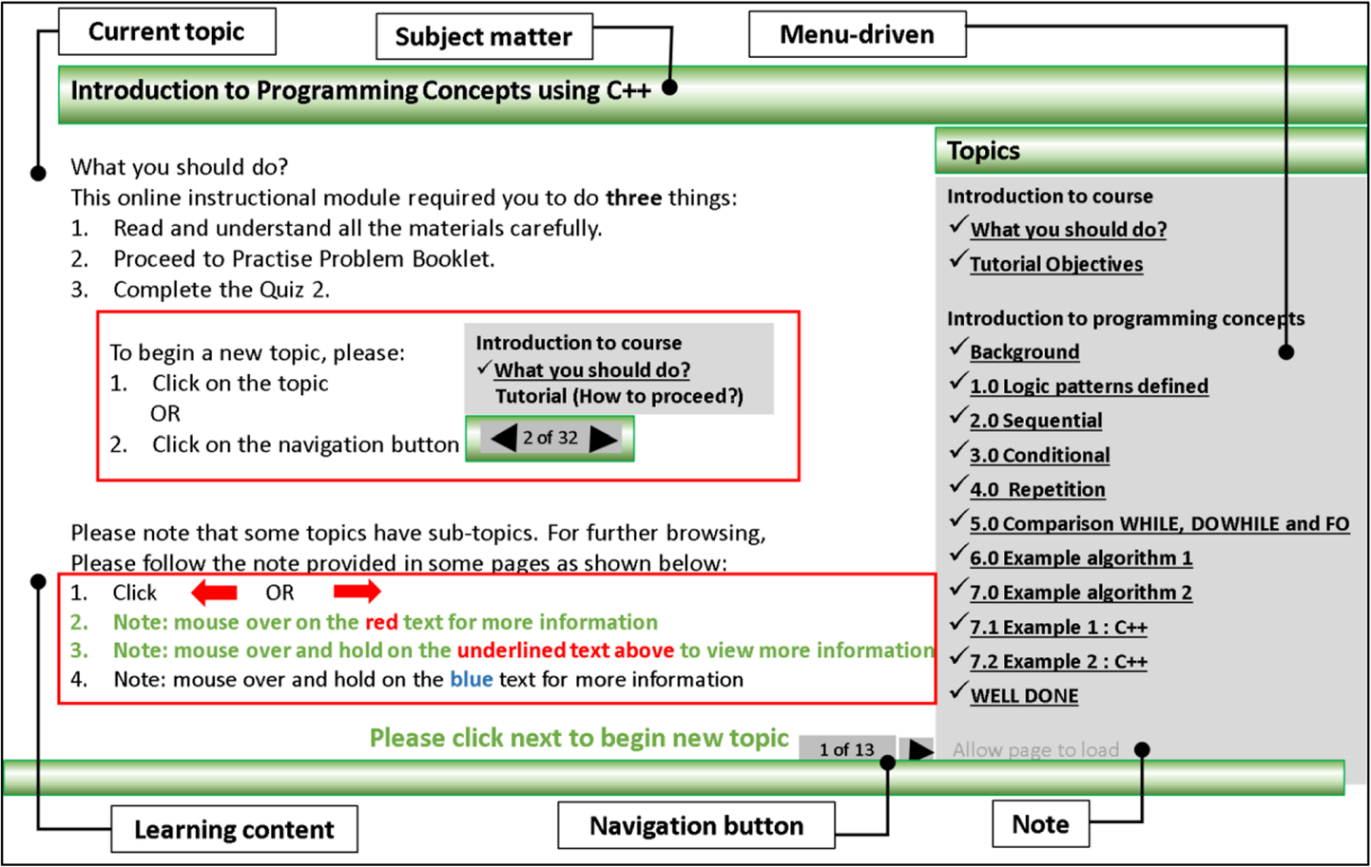
Cognitive performance of ICS groups with T1 and T2 (Anonymous 2012a). This figure shows the results in a graphical representation showing the interactive nature of the cognitive performance of integrated cognitive style (ICS) wholist-verbaliser, wholist-imager, analytic-verbaliser, analytic-imager for the two instructional treatments: T1 (text-plus-textual metaphor) and T2 (text-plus-graphical format) based on average dlv

Figure 10 shows the first screen of the Anonymous (2012a) WMIS. This screen displayed knowledge navigation instruction or guidance on how to work through the instructional modules, thus making navigation of the topics/modules more possible by the menu-driven facility of the (knowledge) navigation buttons or hyperlinks.

A navigation button located at the bottom right of the screen offered ‘previous’ and ‘next’ arrows enabled the learners to move between topics. This type of button also indicated the learners’ current location and the number of remaining topics. The instructional materials were always located on the white background area at the left side of the screen. The instructional materials were presented in dark text on a white background to facilitate ease of reading for learners who may have had colour perception deficiencies (McCracken and Wolfe 2004). To ensure that all information was seen in the one place, no scrolling of the screen was required. Therefore, to reduce extraneous cognitive load during learning, the instructional interface was designed consistently in terms of the usability and relating to the appearance, functionality and instructional ordering. Appearance meant using consistent ‘visual elements’ such as layout, text style, colour and graphics throughout the instructional system. Functionality meant the same ‘(knowledge) navigation element’ such as ‘previous’ and ‘next’ arrows on each screen which allowed the learners to navigate through the topics. Instructional ordering meant the screen organisation remained consistent throughout the WMIS, for instance the navigational button located at bottom right of each screen (Galitz 2007).

#### Research design

The Anonymous (2012a) study involved a quasi-experimental 2 × 4 factorial research design combined with non-equivalent comparison or control group design that was manipulated to observe and analyse the variables. There were two independent variables: (a) ‘instructional strategy’, (T1) text-plus-textual treatment, and the (T2) text-plus-graphical treatment, and (b) ‘cognitive preference’ expressed as a CSA ratio (wholist-analytic/verbaliser-imager). The researcher used the CSA ratio to split the participants with similar ratio results into the instructional treatments (T1 or T2).

#### Methodology

**Fig. 11:**
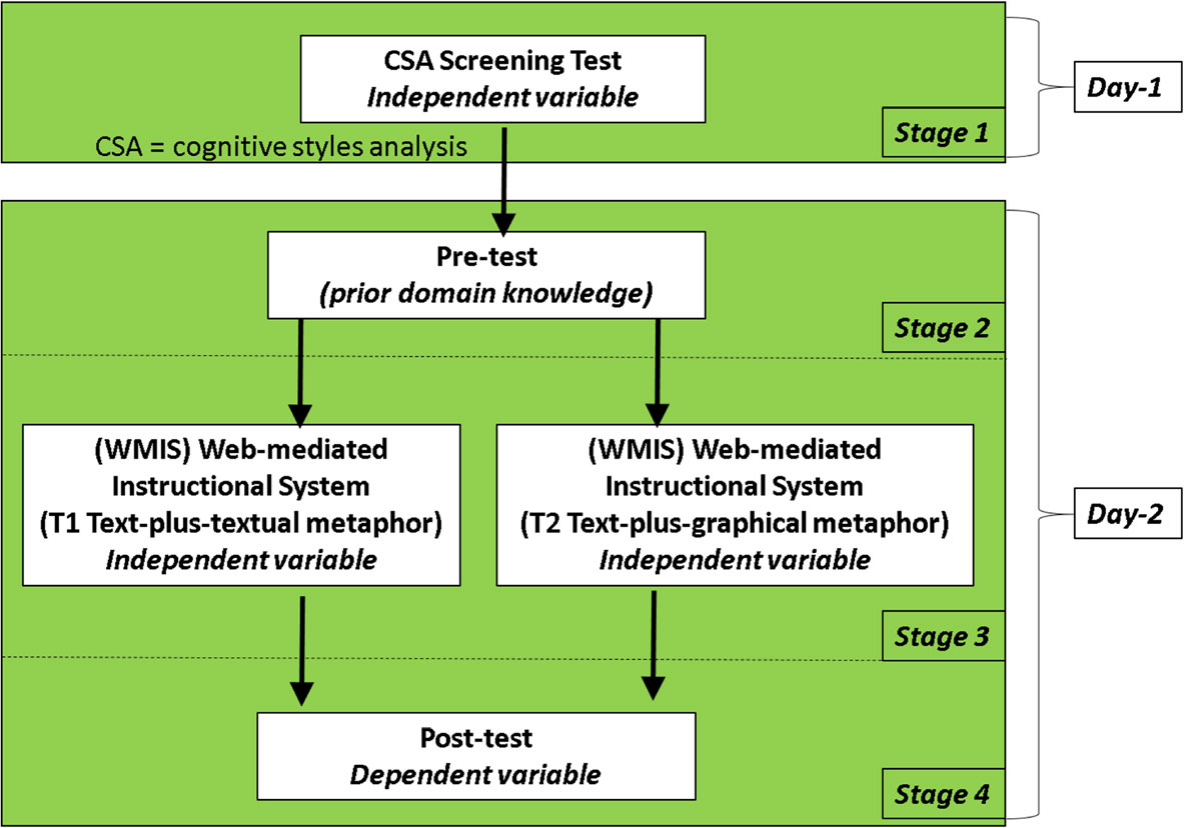
Research schedule (Anonymous 2012a). This figure shows the research schedule comprising the four research study stages: stage 1, day 1—involving the CSA screening test to allocate participants to their instructional treatment; stage 2, day 2—involving the pre-test for prior domain knowledge; stage 3, day 2—involving the experiment; and stage 4, day 2—the post-test

A total of 399 participants were involved in this study. There were four stages involved in this experiment (see Fig. 11). The day 1 activity was carried out well before participants underwent the pre-test. The day 2 activities took place in the students’ eighth week of their normal semester. Starting with a pre-test for prior domain knowledge, there were two (3-h) sessions (T1 and T2), followed by the post-test. The quasi-experiment was conducted during this period because the participants were scheduled to learn the ‘repetition control structure’ topic in their normal classroom tuition.

The usual CSA screening test was conducted in stage 1 as pictured in Fig. 11. On day 2 of the experiment, participants were given the paper-based questionnaire that comprised a 41-item pre-test (stage 2). Immediately after completing the pre-test questionnaire, the participants were required to access their WMIS treatment (stage 3). The duration to access the WMIS was limited to a 1-h period due to the fact that it was replacing their normal 1-h lecture during their tutorial session and as such, they applied what they understood from the WMIS material into the online practice problem booklet. Stage 4 proceeded once they had completed the WMIS material. Therefore, the participants then received a 41-item post-test questionnaire. Because the Rasch analysis does not cope well with absent data, it was important for the participants to provide their best answers. And so, in order to avoid incomplete responses for both the pre- and post-tests, they were guided by written and verbal explanations of the importance of completing all test items.

#### Data analysis

**Fig. 12:**
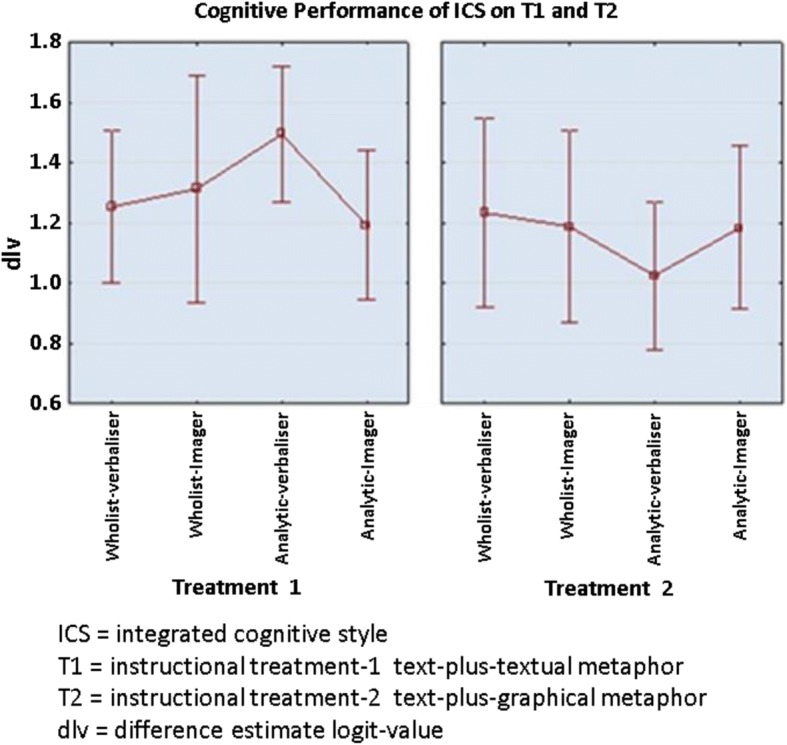
Cognitive performance of ICS groups with T1 and T2 (Anonymous 2012a). This figure shows the results in a graphical representation showing the interactive nature of the cognitive performance of integrated cognitive style (ICS) wholist-verbaliser, wholist-imager, analytic-verbaliser, analytic-imager for the two instructional treatments: T1 (text-plus-textual metaphor) and T2 (text-plus-graphical format) based on average dlv

There were 352 participants that successfully completed the pre- and post-tests. Participants with zero scores and perfect scores were excluded from the analysis. This was because the participants with zero scores provided no evidence of whether any learning had occurred. Similarly, the participants with perfect scores were unable to show how much learning had occurred (Izard 2005). In the final analysis, only 305 participants were counted for the effect size computation. The cognitive performance of students was measured using the difference between pre- (prlv) and post-test (polv) estimate logit value (dlv) rather than just post-test results. The total of N value for the ICS group for T1 was calculated by adding *N* values of all ICS groups (wholist-verbaliser, *N* = 43; wholist-imager, *N* = 29; analytic-verbaliser, *N* = 48; and analytic-imager, *N* = 44), while the total of *N* value for T2 was 141. The graphical presentation in Fig. 12 displays the cognitive performance of integrated cognitive style (ICS) with the text-plus-textual (T1) and text-plus-graphical format (T2) based on average dlv.

Regardless of their cognitive preferences, the students performed better with the T1 than T2. There was an interesting finding with the ICS groups: the analytic-verbaliser group had a significantly higher average on cognitive performance with the T1 than T2. A medium effect size was observed (*d* = 0.62) (Cohen 1977); for the wholist-imagers, there was a negligible effect size observed (*d* = 0.12). An even more trivial effect size was computed for the wholist-verbalisers and analytic-imagers (*d* = 0.02 and *d* = 0.01 respectively).

#### Results

An important practical effect was found with the analytic-verbalisers who performed better with the T1 (text-plus-textual metaphor) than with the T2 (text-plus-graphical metaphor). The analytic-verbaliser group was the top performer when given T1 (mean dlv = 1.49), yet their counterpart was the worst performer with T2 (mean dlv = 1.03). The top performer in T2 was a wholist-verbaliser (mean dlv = 1.23) group. The wholist-imagers performed slightly better in T1 (mean dlv = 1.31) than T2 (mean dlv = 1.19). Further, there was no practical important effect for wholist-verbaliser (mean dlv T1 = 1.25, mean dlv T2 = 1.23) and the analytic-imager (mean dlv T1 = 1.19, mean dlv T2 = 1.18) groups, on both treatments (T1 and T2), as they performed similarly with each treatment, yet the cognitive performance of the wholist-verbaliser group was better than the analytic-imagers on both instructional treatments.

## Discussion

This paper has used three interrelated research studies to address the growing need for confirming whether those working with big data have the sufficient digital skills to cope (problem-solving abstract concepts, paper-based instructional metaphor format and cognitive style, and online instructional metaphors and cognitive style). These experiments showed the progression from the earlier methodological approach used for measuring proficiency between novice and experienced programmers with CCT (more commonly called traditional statistical measures), to adopting a more comprehensive unidimensional scale that empowers comprehension of human performance and test item performance relative to each other. This latter methodology offers an effective tool for understanding individual differences in digital skill development.

The lessons learned from the Bagley (1991) results were that it is important to provide flexible training modules that afford different cognitive skill development pathways for novice and experienced people. The Bagley results confirmed two hypotheses: (1) that giving a structured instructional pedagogy (forced step-by-step skill development) improved the learning of computer programming for people with no prior experiential knowledge of computer programming and (2) that giving a discovery instructional pedagogy (allowing a self-selecting instructional pathway) improved learning for people possessing prior knowledge of computer programming. Moreover, when considering the interactive effect of instructional strategy and prior domain knowledge (experienced/novice) on performance outcomes—given the structured instructional format—the experienced learners did not perform as well.

In terms of managing big data (digital) tasks, it is important to know whether people have acquired problem-solving skills to solve abstract problems. Nevertheless, at the time of the Bagley study, the concept of big data had not yet surfaced. Instead, the term ‘computer literacy’ described the human-computer interaction (HCI). Researchers were mainly interested in the machine-dimension of the HCI (Anonymous 2008) examining peoples’ capabilities expressed as arithmetic reasoning (problem-solving) abilities (Bagley, 1991) to manage the computer operations. It took the advent of Web 2.0 and powerful computers to advance big data platforms that has refocused educational research on how to prepare effective pedagogies to enhance the digital skill development necessary to deal with big data characteristics.

The second study mentioned in this paper involved a much closer examination of the interactivity of preferred cognitive style and a paper-based instructional pedagogy on the Rasch measured performance outcomes. To answer the question, targeted textual pedagogies were replaced with text-plus-textual metaphors (T1)/text-plus-graphical metaphors (T2). The study used the CSA ratio to split the research population according to the Riding and Cheema (1991) cognitive style continua (verbal-imagery (V:I), to depict a representation of information during thinking that may change according to the task at hand (Riding 1993); wholist-analytic (W:A), to depict mode of processing information, which Riding (1993) maintained is inherent and does not change).

To answer the research question (*does the interaction of instructional format and cognitive style construct affect the acquisition of abstract programming concepts?*) surprisingly, the verbalisers performed best when given the instructional format enhanced with graphical metaphors, and some imagers responded better with text-only material. This result was contra to the axiom that assumes verbal preferenced people perform best with textual pedagogies, while imagery preferenced people perform best with pictures.

Examining the interactive effects however of the W:A dimension revealed that the ‘wholists’ performed better with the graphical metaphors than the text only pedagogies. Yet when considering the more detailed analysis of results involving the combined cognitive style analysis (CSA) dimensions involving wholist-verbaliser (W:V), analytic-verbaliser (A:V), wholist-imager (W:I) and analytic-imager (A:I), the explanation was more complex. Consequently, using the Rasch measurement model, this research successfully showed that when given a graphics-enhanced pedagogy, particular combinations of the Riding and Cheema (1991) cognitive style construct had the potential to perform much better than others. Namely the largest effect size for the integrated (combined) cognitive style groupings were the W:I and A:V. This result was also confirmed in the final experiment where the A:I group performed poorly with the graphical pedagogy enhancements.

In terms of managing big data and implications arising from this research are for the need to understand the complexity of how particular people will perform the necessary analytic problem-solving tasks/operations more comfortably than others will.

The third study mentioned above in this paper extended the second (Anonymous 2000a), still examining the interactivity of preferred cognitive style but in a digital (online) instructional pedagogy, again using the Rasch measured performance outcomes. This study interrogated the ICT pedagogical elements, text-plus-textual metaphor (T1) or text-plus-graphical metaphors (T2). These treatments formed the basis of an investigation on whether the interactive effects of the independent variables (the participants’ CSA ratio and instructional treatment) had contributed to the results. In this case the research question was: *does the interactive effect of web-mediated instructional strategies and a learner’s cognitive style preference affect the acquisition of introductory computer programming concepts?*

The research question was answered successfully using the combined CSA ratio. Importantly, the A:V group performed better with the T1 than with the T2. The A:V group was the top performing group when given T1, yet given T2 they were the worst. The top performing group with T2 was the W:V, while the W:I group performed slightly better in T1 than T2. Further, there was no important effect for W:V and the A:I groups on both T1 and T2, as they performed similarly with each treatment, yet the cognitive performance of the W:V group was better than the A:I group on both instructional treatments.

These results further emphasise the importance of knowing whether particular people will perform the necessary analytic problem-solving tasks/operations more comfortably than others will, when required to manage big data.

## Conclusions

The term ‘big data’ means many things to many people, so we say it is something that the average researcher should not be apprehensive to investigate. One way to enter this relatively newly labelled field is to concentrate on managing the development of digital skills that are involved in maintaining big data platforms. And so, to determine correctly an individual’s digital skills’ level is made possible by systematically following the Anonymous (2000a) or the Anonymous (2012a) methodology. Such research projects can achieve similar measurable and valid analysis results that correctly determine an individual’s digital skill level. Firstly, one must define the knowledge levels within the study domain, and then design a test instrument to capture the participant’s skills. Then a structured scoring rubric must be developed to scale participant achievement on the (digital skills) test. The instrument must first be validated, with the poorer test items (too easy, too hard, or poorly worded such that participants cannot understand what is expected) either removed, or modified and re-validated. Once the instrument is fully validated then it can be implemented. This validated test can be used as a one-off skills level bench-mark. However, to measure the effectiveness of an instructional programme, it is recommended that the methodology involves a pre-test to measure participants’ prior domain knowledge, followed by the instructional treatment, and then a post-test to measure what they know (or do not know) after the treatment. The participant performance can then be measured as the difference in QUEST scores between the pre-test and the post-test. The internal consistency of the Rasch IRT model and the statistical variables generated by packages such as the QUEST Interactive Test Analysis System and a newer version called RU2030 (Andrich et al. 1997-2005) to allow the researcher to produce quantitative research results from limited data and fewer participants. Finally, a Cohen statistical power analysis can be used to determine the magnitude of effect of different treatments or different participant characteristics on performance outcomes according to Anonymous (2016).

This paper takes the view initiated by Sun (2018) to convey that managing/working in a big data environment involves a plethora of high-level digital skills that will change according to the cognitive preferences of each individual and the task at hand. Therefore, discerning what people know and what they do not know is imperative. Otherwise, endless mess will result, with ill-informed people blaming ‘the data’ and projects not realising their potential.

## Abbreviations

A:I: Analytict:imager cognitive style dimension
A:V: Analytic:verbaliser cognitive style dimension
ANOVA: Analysis of variance
CSA: Cognitive styles analysis
CTT: Classical test theory
d: Effect size
dlv: Difference logit value
HCI: Human-computer interaction
HE: Higher education
ICS: Integrated cognitive style
ICT: Information communications technology
ID: Instructional design
IRT: Item response theory
IS: Information systems
IT: Information technology
polv: Post-test estimate logit value
prlv: Pre-test estimate logit value
T1: Treatment 1 (text-plus-textual metaphor)
T2: Treatment 2 (text-plus-graphical metaphor)
W:I: Wholist:imager cognitive style dimension
W:V: Wholist:verbaliser cognitive style dimension
WAIS: Weschsler Adult Intelligence Scale
WMIS: Web-mediated instructional system
