# Correction to: Big data management skills: accurate measurement

**DOI:** 10.1186/s41039-018-0074-z

**Published:** 2018-07-03

**Authors:** Elspeth McKay, Marlina Binti Mohamad

**Affiliations:** 1 RMIT University, School of Business Information Technology and Logistics, GPO Box 2476, Melbourne, Victoria 3000 Australia; 2 Department of Engineering Education, Faculty of Technical and Vocational Education, Universiti Tun Hussein Onn Malaysia, 86400 Parit Raja, Batu Pahat, Johor Malaysia

## Correction

Owing to an unfortunate mistake in typesetting, in the original publication of this article (McKay & Mohamad, 2018), the citation and legend of some figures were incorrectly displayed. Besides, anonymous information was embedded in the article by mistake after double blind peer reviewing. We list the errors and corrections below:

Figures citation errors and corrections:

**Table:** 

Figures citation upon publication	Figures citation upon correcting
T1 Fig. 3	T1 Fig. 4
T2 Fig. 4	T2 Fig. 5
Fig. 5	Fig. 6
Figure 6	Figure 3
Fig. 3	Fig. 4
Fig. 4	Fig. 5

Figures legend errors and corrections:

**Table:** 

Figures legend upon publication	Figures legend upon correcting
Fig. 3 Text-plus-textual metaphor (T1)—repetition programming logic (Anonymous 2000a). This figure represents a textual metaphor for a ‘do while loop’	Fig. 3 Common expository instructional format (McKay 2000a p.163) This figure represents an example of an instructional strategy that provides key information of a programming control flow statement – known as a ‘do while loop.’ It is showing the testing condition and the procedure for dealing with a condition that has become false.
Fig. 4 Text-plus-graphical metaphor (T2)—repetition programming logic (Anonymous 2000a). This figure represents an example of a text-plus-graphical metaphor for a ‘do while loop’	Fig. 4 Text-plus-textual metaphor (T1) – repetition programming logic (McKay, 2000a p.165) This figure represents a textual metaphor for a ‘do while loop.’
Fig. 5 QUEST variable map (Anonymous 2000a). This figure shows how the QUEST estimate develops a unidimensional(logit) scale (− 3.0 to 1.0) with equal intervals along each axis as it measures participants’performances and test items together. The x’s on the left hand side of the figure represent an individual participant’s performance with the total number of participants being 195. On the right hand side of the figure is the difficulty rating of each test item’s performance (partial credit scored test items have multiple entries: 8.1, 8.2 and 9.3, 20.2)	Fig. 5 Text-plus-graphical metaphor (T2) – repetition programming logic (McKay, 2000a p.165) This figure represents an example of a text-plus-graphical metaphor for a ‘do while loop.’
Fig. 6 QUEST fit map (Anonymous 2000a). This figure shows the fit statistics (listed horizontally .56 to 1.40 is the infit mean square); the asterisks represent the magnitude of the fit statistic for the test item on the same line. The test items that fall between the two vertical dotted lines (thresholds .77 to 1.30) are considered acceptable; test items to the left overfit (see test item 34), indicating duplication or having limited contribution. Underfit test items to the right of the threshold lines, measure something else and need rewording	Fig. 6 Digital skills acquisition for introductory programming (McKay, 2000a p.175) This figure presents a ‘test instrument specification matrix’ used to design the test-items to determine the expected introductory programming knowledge acquisition.
Fig. 7 Relative distribution—four groups (Anonymous 2000a). This figure shows the relative distribution of the four-instructional treatment/gender groups (treatment 1—textual metaphor and treatment 2—graphical metaphors). Females given the graphical metaphors achieved the highest post-test distribution. Females with the textual metaphor format had the lowest distribution. The two male groupings had similar distributions, resting between the two female distributions	Fig. 7 QUEST variable map (McKay, 2000a p.220) This figure shows how the QUEST estimate develops a uni-dimensional (logit) scale (− 3.0 to 1.0) with equal intervals along each axis as it measures participants’ performances and test-items together. The x’s on the left hand side of the figure represent an individual participant’s performance with the total number of participants being 195. On the right hand side of the figure, is the difficulty rating of each test-item’s performance (partial credit scored test-items have multiple entries: 8.1, 8.2 and 9.3, 20.2).
Fig. 8 First screen of web-mediated instructional module (Anonymous 2012a). This figure shows the opening web-mediated instructional system’s screen display including how to navigate the instructional content and guidance on how to work through the instructional modules, (knowledge) navigation buttons or hyperlinks, and menu positioning relating to the current topic and learning content	Fig. 8 QUEST fit map (McKay, 2000a p.222) This figure shows the fit statistics (listed horizontally .56 to 1.40 is the infit mean square); the asterisks represent the magnitude of the fit statistic for the test-item on the same line. The test-items that fall between the two vertical dotted lines (thresholds .77 to 1.30) are considered acceptable; test-items to the left overfit (see test-item 34), indicating duplication or having limited contribution. Underfit test-items to the right of the threshold lines, measure something else and need rewording.
Fig. 9 Research schedule (Anonymous 2012a). This figure shows the research schedule comprising the four research study stages: stage 1, day 1—involving the CSA screening test to allocate participants to their instructional treatment; stage 2, day 2—involving the pre-test for prior domain knowledge; stage 3, day 2—involving the experiment; and stage 4, day 2—the post-test	Fig. 9 Relative distribution – 4-groups (McKay, 2000a p.235) This figure shows the relative distribution of the four-instructional treatment/gender groups (Treatment-1 textual metaphor and Treatment-2 graphical metaphors). Females given the graphical metaphors achieved the highest post-test distribution. Females with the textual metaphor format had the lowest distribution. The two male groupings had similar distributions, resting between the two female distributions.
Fig. 10 Cognitive performance of ICS groups with T1 and T2 (Anonymous 2012a). This figure shows the results in a graphical representation showing the interactive nature of the cognitive performance of integrated cognitive style (ICS) wholist-verbaliser, wholist-imager, analytic-verbaliser, analytic-imager for the two instructional treatments: T1 (text-plus-textual metaphor) and T2 (text-plus-graphical format) based on average dlv	Fig. 10 First screen of web-mediated instructional module (Mohamad, 2012 p.117) This figure shows the opening web-mediated instructional system’s screen display – including: how to navigate the instructional content and guidance on how to work through the instructional modules; (knowledge) navigation buttons or hyperlinks; and menu positioning relating to the current topic and learning content.
Fig. 11 Research schedule (Anonymous 2012a). This figure shows the research schedule comprising the four research study stages: stage 1, day 1—involving the CSA screening test to allocate participants to their instructional treatment; stage 2, day 2—involving the pre-test for prior domain knowledge; stage 3, day 2—involving the experiment; and stage 4, day 2—the post-test	Fig. 11 Research schedule (Mohamad, 2012 p.104) This figure shows the Research Schedule comprising the four research study stages: Stage-1, Day-1 involving the CSA screening test to allocate participants to their instructional treatment; Stage-2, Day2 involving the pre-test for prior domain knowledge; Stage-3 Day-2 involving the experiment; and Stage-4 Day-2 the post-test.
Fig. 12 Cognitive performance of ICS groups with T1 and T2 (Anonymous 2012a). This figure shows the results in a graphical representation showing the interactive nature of the cognitive performance of integrated cognitive style (ICS) wholist-verbaliser, wholist-imager, analytic-verbaliser, analytic-imager for the two instructional treatments: T1 (text-plus-textual metaphor) and T2 (text-plus-graphical format) based on average dlv	Fig. 12 Cognitive performance of ICS groups with T1 and T2 (Mohamad, 2012 p.177) This figure shows the results in a graphical representation of the interactive nature of the cognitive performance of integrated cognitive style (ICS) wholist-verbaliser, wholist-imager, analytic-verbaliser, analytic-imager for the two instructional treatments: T1 (text-plus-textual metaphor) and T2 (text-plus-graphical format) based on average dlv.

Anonymous information errors and corrections:

**Table:** 

Blinded citations upon publication	Blinded references upon publication	Unblinded citations upon correcting	Unblinded references upon correcting
Anonymous 1999a	Anonymous (1999a). Details omitted for double-blind reviewing.	McKay 1999a	McKay, E. (1999a). Exploring the effect of graphical metaphors on the performance of learning computer programming concepts in adult learners: A pilot study. *Educational Psychology, 19*(4), 471–487.
Anonymous 1999b	Anonymous (1999b). Details omitted for double-blind reviewing.	McKay 1999b	McKay, E. (1999b). An investigation of text-based instructional materials enhanced with graphics. Educational Psychology, 19(3), 323–335.
Anonymous 2000a	Anonymous (2000a). Details omitted for double-blind reviewing.	McKay 2000a	McKay, E. (2000a). Instructional strategies integrating the cognitive style construct: A meta-knowledge processing model (contextual components that facilitate spatial/logical task performance). Com. Sci. & Info. Sys.(Ph. D. thesis – Deakin University, Geelong).
Anonymous 2000b	Anonymous (2000b). Details omitted for double-blind reviewing.	McKay 2000b	McKay, E. (2000b). Measurement of Cognitive Performance in Computer Programming Concept Acquisition: Interactive effects of visual metaphors and the cognitive style construct. Journal of Applied Measurement, 1(3), 257–286.
Anonymous 2008	Anonymous (2008). Details omitted for double-blind reviewing.	McKay 2008	McKay, E. (2008). *The Human-Dimensions of Human-Computer Interaction: Balancing the HCI Eq.* (1 ed. Vol. 3). Amsterdam, Netherlands: IOS Press.
Anonymous 2012a	Anonymous (2012a). Details omitted for double-blind reviewing.	Mohamad 2012	Mohamad, M. (2012). The effects of Web-mediated instructional strategies and cognitive preferences in the acquisition of introductory programming concepts: A Rasch model approach. Doctoral Thesis RMIT University, School of Business Information Technology and Logistics, Melbourne. https://researchbank.rmit.edu.au/view/rmit:160201/Mohamad.pdf
Anonymous 2012b	Anonymous (2012b). Details omitted for double-blind reviewing.	Alwi and McKay 2012	Alwi, A. & McKay, E. (2012). Consideration for cognitive preferences to enhance effective HCI in online exhibits. International Journal of Computer Information Systems and Industrial Management Applications (IJCISIM), ISSN:2150–7988, 3, 472–479.
Anonymous 2015	Anonymous (2015). Details omitted for double-blind reviewing.	McKay and Izard 2015	McKay, E. & Izard, J.F. (2015, 21–23 July). Evaluate online training Effectiveness: Differentiate what they do and do not know. Paper presented at the 8th International Conference on ICT, Society and Human Beings 2015 (Multi conference on computer science and information systems - MCCSIS), Las Palmas de Gran Canaria. 35–44.
Anonymous 2016	Anonymous (2016). Details omitted for double-blind reviewing.	Barefah and McKay 2016	Barefah, A, & McKay, E. (2016). Evaluating the design and development of an adaptive e-Tutorial module: A Rasch measurement approach. *Paper presented at the Educational Technologies 2016 (ICEduTech)*, RMIT University, Melbourne. https://eric.ed.gov/?id=ED571591

The publisher apologizes to the readers and authors for the inconvenience.

The original publication has been corrected.
